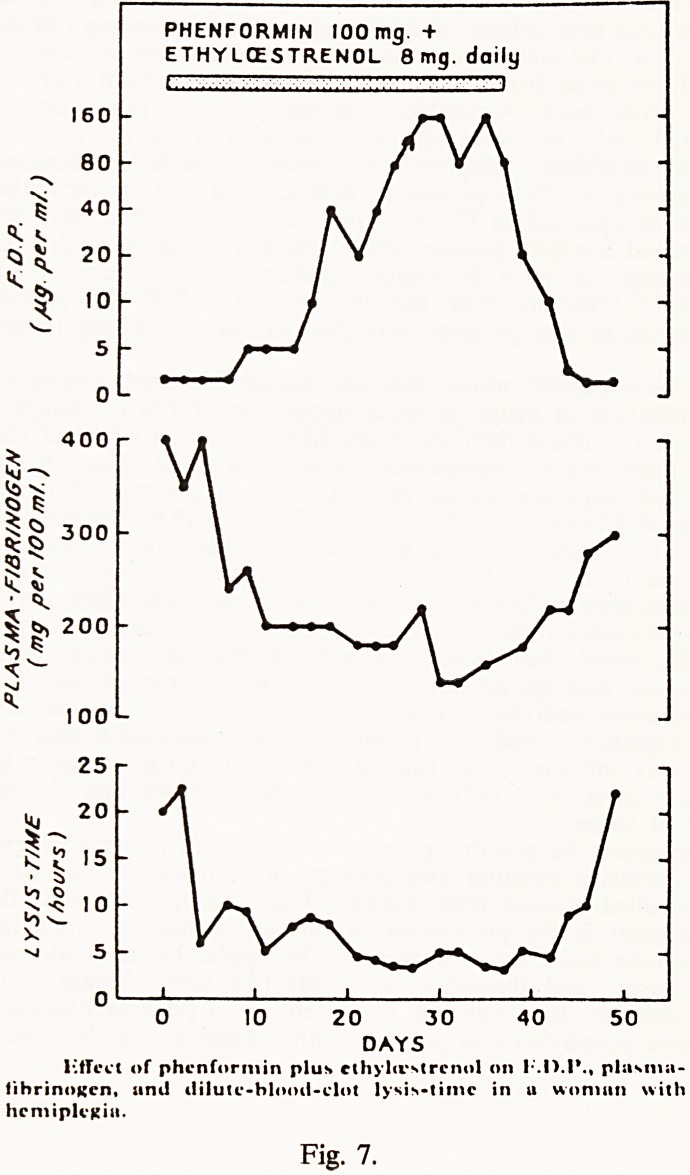# Pharmacological Fibrinolysis

**Published:** 1969-10

**Authors:** G. R. Fearnley


### PHARMACOLOGICAL FIBRINOLYSIS

**G. R. Fearnley**

Until fairly recently, arteriosclerosis and its complications were philosophically accepted as an inevitable consequence of ageing; but the steady increase of coronary deaths in particular, and the equally steady decrease in the age of onset have put an end to such laissez-faire complacency. To pin-point the epidemic acceleration of coronary deaths, a senior colleague told me that when he took the membership in 1927 there was a question on coronary thrombosis, and surprising as it may seem a good many of the candidates had not heard of the condition.

Now, whilst it is difficult to see the wood for the trees, what we are witnessing may be an increase more of vascular occlusion than of arteriosclerosis. William Heberden described angina pectoris 180 years ago, and 19th century pathologists were well aware of arteriosclerosis in all its forms. And yet myocardial infarction escaped recognition in this country until 1925 (Gibson, 1925; McNee, 1925). Or did it? Perhaps before then it was not at all common. It seems unlikely that the excellent morbid histologists at the turn of the century would have missed it if it were occurring with any frequency.

If this be true, despite the multifactorial nature of the problem, perhaps we should be concerned primarily with the prevention of thrombosis, in other words with the circulating fluid, the blood. The situation, however, remains multifactorial because it involves at least three factors, platelet behaviour, coagulation, and last, though I hope not least, fibrinolysis. It is with fibrinolysis that I have been especially concerned, and particularly with its enhancement by drugs given orally, which also influence platelet stickiness, plasma fibrinogen level, and serum cholesterol, thus, as it were, killing several associated birds with one stone.

$$\text{Activator} + \text{plasminogen} \longrightarrow \overset{\overset{\text{Antiplasmin}}{\downarrow}}{\text{plasmin}} + \frac{\text{fibrinogen}}{\text{fibrin}} \longrightarrow \text{Split products}$$

The basis of the fibrinolytic system is an inactive enzyme precursor, plasminogen, which is present in the beta-globulin fraction of plasma. Plasminogen can be converted to the active proteolytic enzyme, plasmin, by a number of activators, some of which like streptokinase are of exogenous origin and others of which are endogenous. As my colleagues and I showed about 16 years ago (Fearnley and Tweed, 1953) normal blood contains an activator of plasminogen which had hitherto escaped detection because it is labile in anticoagulated blood at room temperature (Fearnley *et al.*, 1952). This activator is normally not present in sufficient amount to lyse native or unaltered blood clot *in vitro,* and the reason for this is the presence of antiplasmin or plasmin inhibitor which is present in excess and neutralises any plasmin liberated.

If, however, freshly obtained blood is immediately cooled and diluted one part in ten in phosphate buffer and clotted in the cold with thrombin, such clots will lyse within a few hours of incubation. The reason for this is that dilution differentially reduces inhibition in favour of fibrinolysis; in other words the effect of antiplasmin is diluted out, thus allowing plasmin conversion by the natural activator, and hence lysis of the clot. The low-temperature technique between venepuncture and clot formation is necessary because of the lability of plasminogen activator in blood removed from the body.

Labile activator
↓ Adsorption
Fibrin (plasminogen ⟶ plasmin) ⟶ split products

When blood clots, however, activator is stabilised by absorption to fibrin.

In 1953 I found that clots made from non-fibrinolytic plasma, after being steeped in fresh plasma at low temperature and then washed, showed superficial lysis on incubation, indicating that activator present in the fresh plasma had been absorbed to the clots and thereafter had converted their contained plasminogen to plasmin with consequence lysis (Fearnley, 1953). This led to the concept of fibrinolysis by adsorption (Fearnley, 1961) to explain how the amount of plasminogen activator present in normal blood, which is insufficient to lyse native blood-clot *in vitro* may function as a fibrin-clearing and hence antithrombotic mechanism *in vivo.* Since plasmin attacks fibrinogen and fibrin impartially such a mechanism could provide for lysis of fibrin *in vivo* without impairing haemostasis.

Fig. 1. In a mural thrombus (*a*) or a retracted thrombus (*b*) activator is available for adsorption and concentration from circulating blood. In occlusive thrombosis (*c*) the circulation ceases and activator is not available. (From Fearnley, G. R. (1964). *Brit. med. Bull.*, **20,** 185.)

Although the endothelium of veins is rich in plasminogen activator (Todd, 1959) and indeed may be the principal source of the activator content of circulating blood (Fearnley, 1965), the endothelium of arteries seems to be relatively deficient in activator which is located more in the adventitia of these vessels. If fibrinolysis occurs on the arterial side of the system, in other words if fibrin deposits are removed by this means, adsorption of the activator from circulating blood may be of considerable importance. By such a mechanism the large reservoir of plasminogen activator present in circulating blood could be concentrated at sites of fibrin deposition with consequently far more rapid and effective lysis than is seen in the test-tube. In this way fibrinolysis may function as a fibrin-clearing mechanism without impairing haemostasis, for as shown in Fig. 1 an occlusive thrombus halts adsorption of activator.

Before discussing fibrinolysis in occlusive vascular disease and its enhancement with drugs given by mouth, may I briefly outline the two methods we use for measuring spontaneous fibrinolysis? The first of these, the dilute blood clot lysis-time (Fearnley *et al.*, 1957, Fearnley, 1964) is our standby. It is the time required for lysis at 37°C of clots made with thrombin, of freshly obtained blood diluted one part in ten in phosphate buffer, pH 7.4. The lysis-time, which is the time from incubation to the break-up of the clot, lies between 1½ and 7 hours for normal people. Lysis-time and fibrinolytic activity are, of course, inversely related, so that a long lysis-time means low fibrinolytic activity and a short lysis-time means high activity. Long lysis-times are obtained by photographic recording, and normally we do not record beyond 24 hours.

Secondly, if the euglobulin fraction of plasma is precipitated, anti-plasmin remains in the supernatant. The euglobulin fraction can then be suspended in buffer, clotted with thrombin, incubated at 37°C, and observed for lysis. This constitutes another method of measurement, the euglobulin lysis-time. Normal lysis-times by this method range between 1-2 hours; and the results of this test in general correlate well with the dilute blood clot lysis-time when both methods are performed on the same blood sample. The ratio of about 3:1 between the times in the two methods is accountable to the fact that in the blood clot lysis-time antiplasmin is still present, its effect being diminished but not removed by dilution, whereas in the euglobulin method antiplasmin is absent.

If we postulate that natural fibrinolysis is concerned with the patency of blood vessels, the first question we have to ask is: Do people with ischaemic conditions differ from healthy people in respect of blood fibrinolytic activity? This is not so easy to answer as one might think, because occlusive vascular disease is now the commonest cause of death, and in any age-matched series of patients and controls an unknown proportion of the controls will have imminent or undisclosed occlusive vascular disease.

Table I shows the results of 3 determinations of the dilute blood clot lysis-time in 107 male survivors of myocardial infarction and 90 healthy men of similar ages with normal electrocardiograms. Taking a lysis-time of 7 hours or longer (i.e. 7-24 hours) on two or more of the three tests to indicate low fibrinolytic activity, it can be seen that fibrinolysis was defective in

32% of the patients compared with 12% of the controls. When the findings are viewed in relation to age, it can be seen that the incidence of defective fibrinolysis among the coronary patients diminshed with age, until after the age of 60 it was virtually the same as that among the controls. One possible explanation for this is that coronary artery disease plus low fibrinolytic activity carries a poor expectation of survival; in other words such patients tend to be eliminated by natural selection (Chakrabarti *et al.*, 1968). To establish this would require a prospective follow-up of patients with coronary artery disease whose fibrinolytic activity is known, and we have started such a study.

Turning to occlusive vascular disease of the lower limb, Nestel (1959) compared 30 males suffering from intermittent claudication with 30 age-matched healthy men by means of a single determination of the dilute blood clot lysis-time. The mean lysis-time of the controls, 4.9 hours, was of the same order as we and others have found in healthy males, whereas that of the claudicators, 13.3 hours, was well into the range of low fibrinolytic activity. Our own studies of repeated measurements on patients with occlusive vascular disease of the lower limb indicate that 60% of them have lysis-times persistently longer than 7 hours (Fearnley and Chakrabarti, 1964).

There is therefore evidence of an association between defective fibrinolysis and occlusive vascular disease in the heart and lower limb. That it is causal cannot of course be assumed, but this could be put to the test by therapeutic trial if it were possible to increase fibrinolytic activity pharmacologically in patients with ischaemic conditions. During the past 10 years we have been seeking to achieve this, and we think we now have a means of doing so. As a result of experiments with insulin which has a biphasic influence on fibrinolysis (Fearnley *et al.*, 1959) we studied the effect of the sulphonylureas tolbutamide and chlorpropamide on fibrinolytic activity.

Both drugs were found to increase fibrinolytic activity in in-patients who were studied in the fasting resting state. Fig. 2 shows reduction of the blood clot lysis-time by tolbutamide in a woman with ischaemic disease. It should be noted that there was no appreciable reduction of blood glucose, suggesting that the effect of tolbutamide on fibrinolysis was not a reflection of hypoglycaemia; and also that resistance developed to the fibrinolytic effect of this drug. Similar results were obtained with chlorpropamide but it soon became apparent that resistance to the fibrinolytic effect of the sulphonylureas develops after 3-4 weeks treatment. Nevertheless these were the first drugs found to be capable of increasing fibrinolytic activity for several weeks when given by mouth (Fearnley *et al.*, 1960).

TABLE I

**Incidence of Low Fibrinolytic Activity in Patients and Controls**

| Age-group (yr.) | No. of subjects with B.L.T. of >7 hr. in at least two out of three tests | | chi[2] |
|---|---|---|---|
| | Controls | C.D. Survivors | |
| Under 50 ... | 7 (*19*%) | 17 (*52*%) | 6.46* |
| 50-59 ... ... | 2 (*7*%) | 13 (*34*%) | 5.88* |
| 60 and over ... | 2 (*8*%) | 4 (*11*%) | 0 |
| All ages ... ... | 11 (*12*%) | 34 (*32*%) | — |

* Significant (P=0.05).

Fig. 2. **Increase of fibrinolytic activity by tolbutamide in a woman with status anginosus.**

Fig. 3.

Since the fibrinolytic effect of the sulphonylureas appeared to be independent of hypoglycaemia, it seemed possible that phenformin, which does not reduce the blood glucose levels of normoglycaemic people, might have a fibrinolytic effect, and we tried out this drug as long ago as 1960. Further studies indicated that the effect of phenformin on fibrinolysis, unlike that of the sulphonylureas, was still present at the end of 3 months treatment (Fearnley and Chakrabarti, 1964), but at that time the drug, available only in tablet form, caused such a high incidence of gastro-intestinal intolerance that we had to abandon it for the time being. When, however, phenformin became available in timed-release capsules, which were much better tolerated than the tablets, we were able to use it again. In the meantime, the allied substance, metformin, was found to have a similar fibrinolytic effect (Chakrabarti *et al.*, 1965), and it seemed possible that both drugs might exercise a continuing effect on fibrinolysis in contrast to the resistance which developed in this respect to the sulphonylureas. In 1964, therefore, we began trials of both drugs in two groups of 18 patients.

Before describing the results obtained, I must mention that we had found that testosterone given by injection in large dosage caused a marked increase of blood fibrinolytic activity (Fearnley and Chakrabarti, 1962) and this led us to evaluate anabolic steroids given by mouth for possible fibrinolytic effects. Positive results were obtained, but as with the sulphonylureas resistance developed within a few weeks of treatment (Fearnley and Chakrabarti, 1964).

Fig. 4.

Returning to the trials in two groups of 18 patients, Fig. 4 shows the mean serum cholesterol, plasma fibrinogen, dilute blood clot lysis-time and euglobulin lysis-time in the group who received atromid followed by metformin. It can be seen that atromid gave a temporary reduction of the blood clot lysis-time, and a more sustained reduction of the euglobulin lysis-time. Metformin reduced both lysis times, but after 4 months' treatment both showed some escape indicating that partial resistance was developing. This was very disappointing, for as you will see shortly in the other group of patients, similar resistance was developing to phenformin. At this point we wondered whether a combination of fibrinolytic drugs might be more effective than any drug given singly, and we decided to give ethyloestrenol in a dosage of 4 mg. twice daily to both groups of patients in addition to the metformin or phenformin they were already receiving. I must emphasize that ethyloestrenol, despite its name, is not an oestrogen but an anabolic steroid of progestogen type with mildly androgenic properties. It can be seen that metformin plus ethyloestrenol gave a sustained reduction of both lysis-times for as long as both drugs were given, that is for 12 months; that when metformin was withdrawn and the patients received ethyloestrenol alone both lysis-times escaped, but that when both drugs were given again for the last 6 months of the trial the full fibrinolytic effect was restored. 80% of these patients showed a reduction of both lysis-times during combined treatment. It should be noted that plasma fibrinogen was reduced by metformin plus ethyloestrenol, but that serum cholesterol levels rose.

Fig. 5.

Fig. 5 shows the results obtained in the other group of 18 patients, who were initially treated with metformin followed by phenformin. Partial resistance developed to the fibrinolytic effects of both drugs after 3-4 months treatment, but when phenformin plus ethyloestrenol was given for 12 months a sustained fibrinolytic effect was obtained. Again when ethyloestrenol alone was given there was escape, with restoration of the full fibrinolytic effect when both drugs were given again. 89% of these patients gave a fibrinolytic response. Plasma fibrinogen levels were reduced, and in contrast to metformin plus ethyloestrenol, phenformin plus ethyloestrenol caused a reduction of mean serum cholesterol of about 15%. This investigation therefore showed that the combination of a biguanide with an anabolic steroid would give a sustained increase of fibrinolytic activity in a majority of arteriopathic patients (Fearnley *et al.*, 1967). The phenformin combination was marginally superior to the metformin combination in respect of fibrinolysis and of reduction of plasma fibrinogen; but the two combinations had opposite effects on serum cholesterol, that of phenformin being favourable and that of metformin being unfavourable.

**Mean plasma-fibrinogen, platelet stickiness, and B.L.T. of patients 1-10.**

Fig. 6.

Having found that phenformin plus ethyloestrenol has a favourable influence on fibrinolysis, plasma fibrinogen, and serum cholesterol, we wondered whether it might influence platelet stickiness. This combination of drugs was therefore given to 20 patients with occlusive vascular disease and was found to reduce platelet stickiness to glass by 50% or more in 15 of them (Chakrabarti and Fearnley, 1967). Fig. 6 shows the mean change in platelet stickiness in 10 of the patients who responded in this respect, together with reduction of their mean blood clot lysis-time and plasma fibrinogen level. Neither drug given separately had this effect, and how these drugs reduce platelet stickiness is unknown. The effect appears to be independent of their influence on fibrinolysis and plasma fibrinogen, for in a cross-over trial in 10 patients, metformin plus ethyloestrenol to our surprise was found to increase platelet stickiness in contrast to its reduction by phenformin plus ethyloestrenol. This cross-over study also confirmed the opposite effects of metformin and phenformin on serum cholesterol. It has been possible to follow the effect of phenformin plus ethyloestrenol on platelet stickiness in 12 of our original patients for 14-24 months from the beginning of treatment, and in all the effect has been sustained (Fearnley and Chakrabarti, 1968).

In a study of 8 patients atromid-S (that is clofibrate without androsterone) had an effect on platelet stickiness which was not sustained, and prolonged the dilute blood clot lysis-times of 5 of them, indicating that the drug has antifibrinolytic properties. The differing effects of atromid and atromid-S on fibrinolysis we attribute to the absence of androsterone from atromid-S which behaved like an anabolic steroid in the original atromid (Chakrabarti and Fearnley, 1968).

TABLE II

| | Fibrinolytic Activity | Platelet Stickiness | Plasma Fibrinogen | Serum Cholesterol |
|---|---|---|---|---|
| Metformin + ethyloestrenol | + | − | + | − |
| Clofibrate | − | ± | + | + |
| Phenformin + ethyloestrenol | + | + | + | + |

Effects of drugs currently available on 'thrombogenic factors' and serum cholesterol. Plus=favourable; Minus=adverse; Plus-minus=temporarily favourable.

Table II compares the two biguanide combinations with each other and with atromid-S in respect of the measurements described. Phenformin plus ethyloestrenol seems to us on theoretical grounds to be the agent of choice for trial as a long-term treatment in survivors of vascular occlusions; and we have a pilot study in progress in 50-odd coronary survivors who at the present time have been treated for 1-3½ years. A sustained fibrinolytic response has been obtained in about 90% of those whose fibrinolytic activity was in the low or medium range before treatment was started.

It may be argued that all we have shown so far is alteration of highly artificial *in vitro* tests. Is there any evidence that these drugs have a corresponding effect *in vivo*? Until very recently we could not have answered this question. However, the development of a method for measuring fibrin degradation products in serum has recently afforded us the opportunity to do so. Without going into too much detail, it is now possible to measure the quantity of breakdown products of fibrinogen or fibrin in serum by a tanned red cell technique involving inhibition of haemagglutination. The results are expressed as fibrin degradation products or F.D.P. in micrograms per millilitre of serum. Our range for healthy people with this test is 1.25 to 5 micrograms per millilitre of serum.

Effect of phenformin plus ethylœstrenol on F.D.P., plasma-fibrinogen, and dilute-blood-clot lysis-time in a woman with hemiplegia.

Fig. 7.

Fig. 7 shows the effects of phenformin plus ethyloestrenol on F.D.P., plasma fibrinogen, and the dilute blood clot lysis-time, of a woman studied one month after the onset of a cerebral thrombosis. It can be seen that the earliest effect was a marked increase of fibrinolytic activity followed by a 50% reduction of the plasma fibrinogen, followed by a sharp rise of F.D.P. from less than 5 ug/ml. to reach a peak of 160 ug/ml. These findings provide the first direct evidence that enhancement of fibrinolytic activity by phenformin plus ethyloestrenol, as judged by an *in vitro* test, is accompanied by an increased breakdown of fibrin/fibrinogen in the body. They also confirm that the dilute blood clot lysis-time reflects the behaviour of a functioning fibrinolytic mechanism *in vivo*. The method of assay does not determine whether the degradation products arise from fibrin on the one hand, from fibrinogen on the other, or from both. Available evidence indicates that fibrinogenolysis is encountered only in states of pathological fibrinolytic activity, and that activity of physiological degree, with which we are here concerned, is insufficient to overcome the antiplasmin defence and is thus incapable of lysing fibrinogen as opposed to fibrin. Admittedly, phenformin plus ethyloestrenol reduces raised levels of plasma fibrinogen, but this may be due to depression of biosynthesis or to a feed-back mechanism from removal of fibrin. It seems likely therefore that the increase of F.D.P. by phenformin plus ethyloestrenol in our patients was due to lysis of fibrin (Fearnley *et al.*, 1969).

If this be accepted, more than one possibility needs considering. Should this combination of drugs promote deposition of fibrin (though there is no evidence that it does) then increased fibrinolytic activity and rise of F.D.P. could be homeostatic consequences of such an effect. Were this so, however, the expected sequence would be reduction of plasma fibrinogen followed by increased fibrinolytic activity and rise of F.D.P. Despite some overlap the general tendency in our patients has been for increased fibrinolytic activity, and usually for the increase of F.D.P., to antedate or to accompany reduction of plasma fibrinogen. We think it likely therefore that phenformin plus ethyloestrenol removes deposited fibrin by increasing fibrinolytic activity, in other words that these drugs achieve pharmacological defibrination. If this is so, the findings do not of course establish the site of fibrin removal, though vascular endothelium seems one likely place. At the same time we cannot altogether exclude the possibility that phenformin plus ethyloestrenol in some way influences the rate of fibrinogen/fibrin turnover in the body, and here studies with radioactive fibrinogen before and during treatment might be of value.

In conclusion, the possible prophylactic value of pharmacological defibrination in occlusive vascular and perhaps other disorders can be settled only by a controlled clinical trial. Finally, I must emphasize that the object of such treatment is the prevention of further occlusions, since the effects of the drugs take too long to develop to be applicable to acute situations, for which "arvin" and thrombolytic agents like streptokinase would seem to be more suitable. In a nutshell, the intention of pharmacological fibrinolysis is long-term prophylaxis in patients with occlusive vascular disease.

**References**

Chakrabarti, R., Hocking, E. D. and Fearnley, G. R. (1965) Lancet, *ii*, 256.

Chakrabarti, R., and Fearnley, G. R. (1967) Lancet, *ii*, 1012.

Chakrabarti, R., Hocking, E. D., Fearnley, G. R., Mann, R. D., Attwell, T. N., and Jackson, D. (1968) Lancet, *i*, 987.

Chakrabarti, R. and Fearnley, G. R. (1968) Lancet, *ii*, 1007.

Fearnley, G. R. (1953) Nature (Lond.) *172*, 544.

Fearnley, G. R. (1961) Lancet, *i*, 992.

Fearnley, G. R. (1964) J. Clin. Path., *17*, 307.

Fearnley, G. R. (1965) " Fibrinolysis " Edward Arnold, Lond., p. 56.

Fearnley, G. R., Revill, R. and Tweed, J. M. (1952) Clin. Sci., *11*, 309.

Fearnley, G. R. and Tweed, J. M. (1953) Clin. Sci., *12*, 81.

Fearnley G. R., Balmforth, G. V. and Fearnley, E. (1957) Clin. Sci., *16*, 645.

Fearnley, G. R., Vincent, C. T. and Chakrabarti, R. (1959) Lancet, *ii*, 1067.

Fearnley, G. R., Chakrabarti, R. and Vincent, C. T. (1960) Lancet, *ii*, 622.

Fearnley, G. R., and Chakrabarti, R. (1962) Lancet, *ii*, 128.

Fearnley G. R., Chakrabarti, R. (1964) J. Clin. Path., *17*, 328.

Fearnley, G. R., Chakrabarti, R., and Hocking, E. D. (1967) Lancet, *ii*, 1008.

Fearnley, G. R., and Chakabarti, R. (1968) Lancet, *ii*, 1004.

Fearnley, G. R., Chakabarti R., and Evans, J. F. (1969) Lancet, *i*, 910.

Gibson, A. G. (1925) Lancet, *ii*, 1270.

McNee, J. W. (1925) Quart. J. Med., *19*, 44.

Nestol, P. J. (1959) Lancet, *ii*, 373.

Todd, A. S. (1959) J. Path. Bact., *78*, 281.

## Figures and Tables

**Fig. 1. f1:**
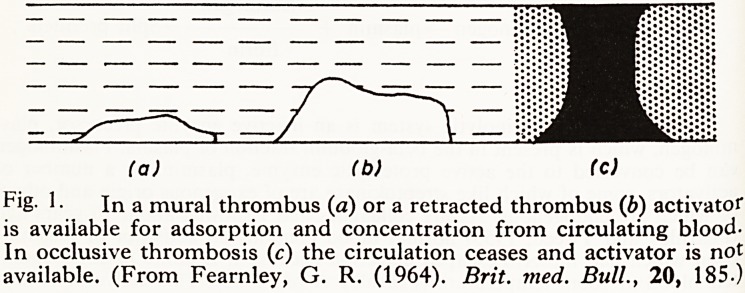


**Fig. 2. f2:**
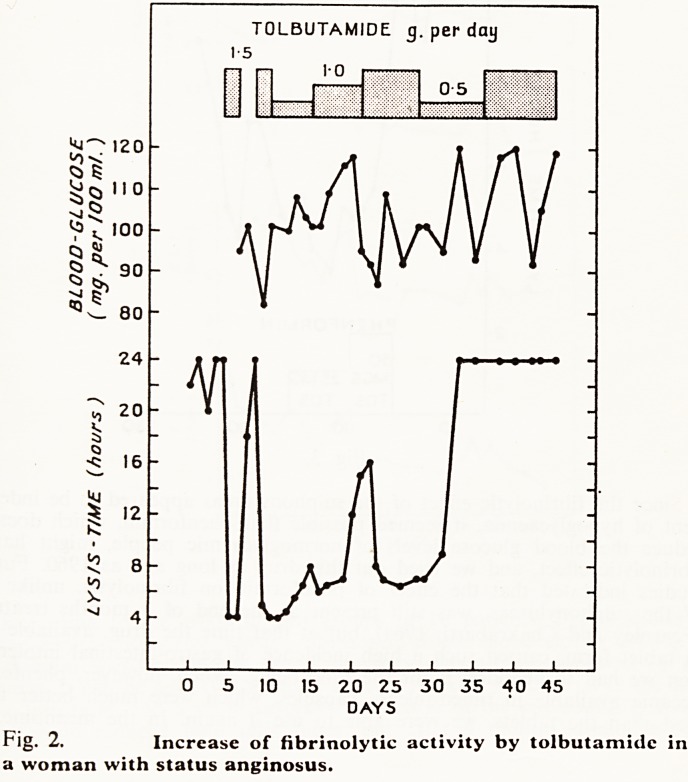


**Fig. 3. f3:**
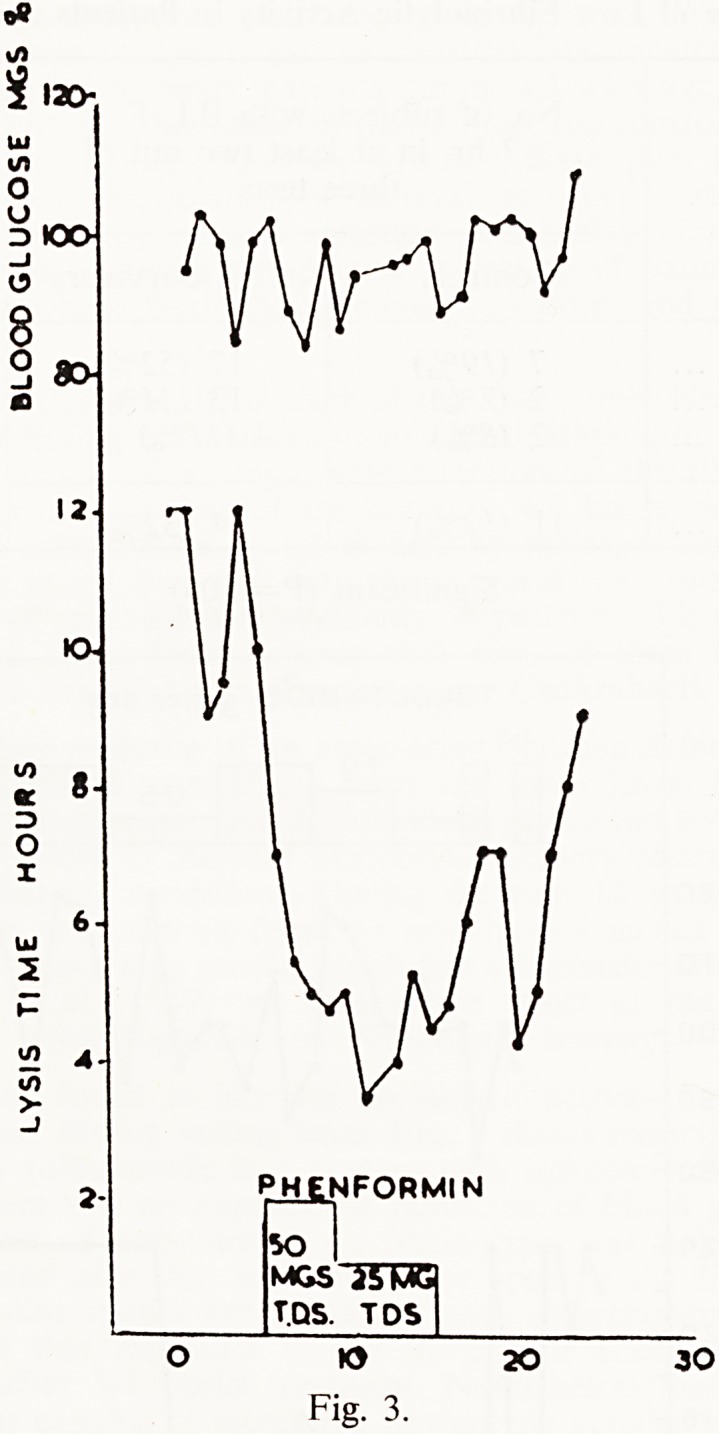


**Fig. 4. f4:**
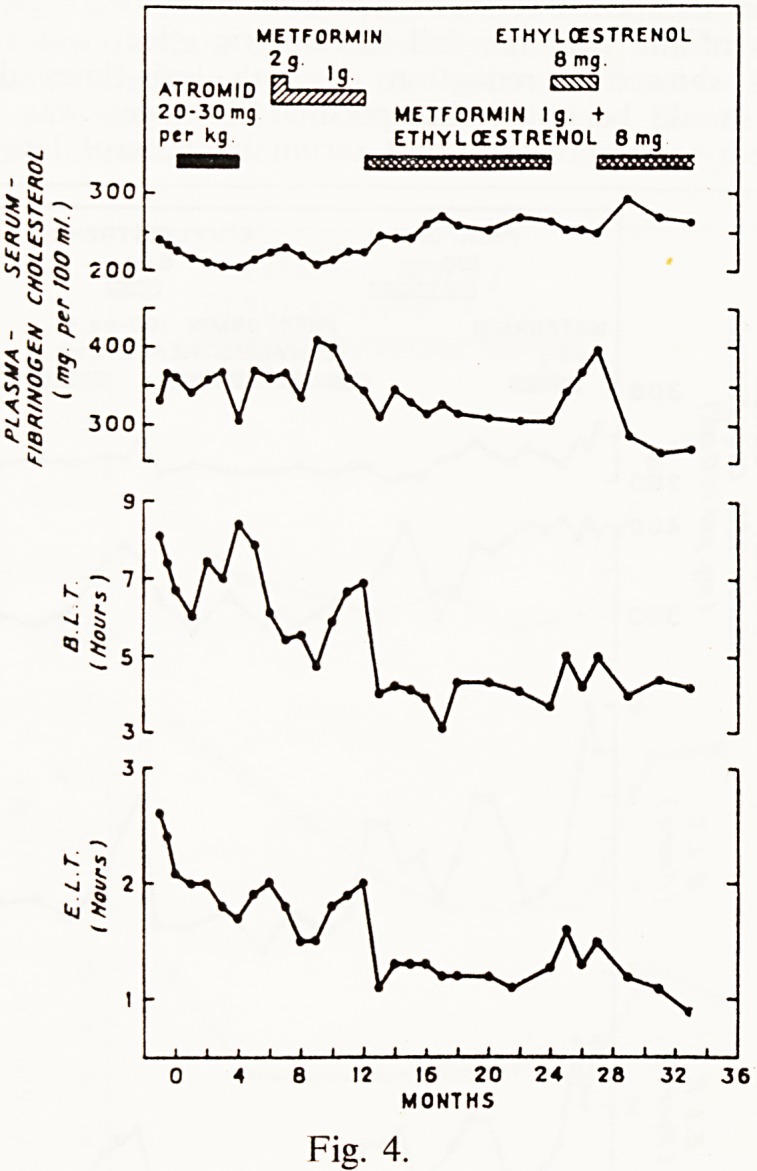


**Fig. 5. f5:**
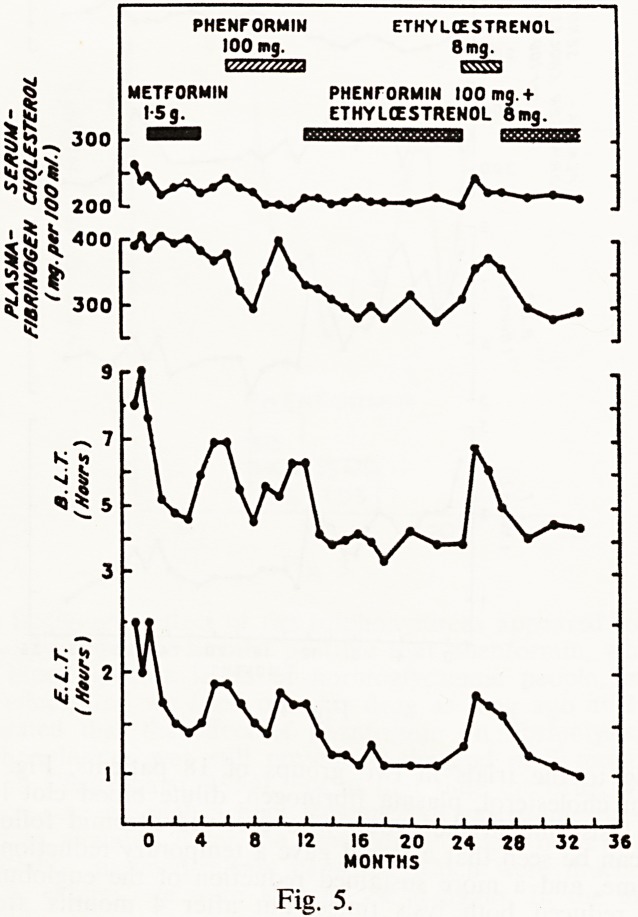


**Fig. 6. f6:**
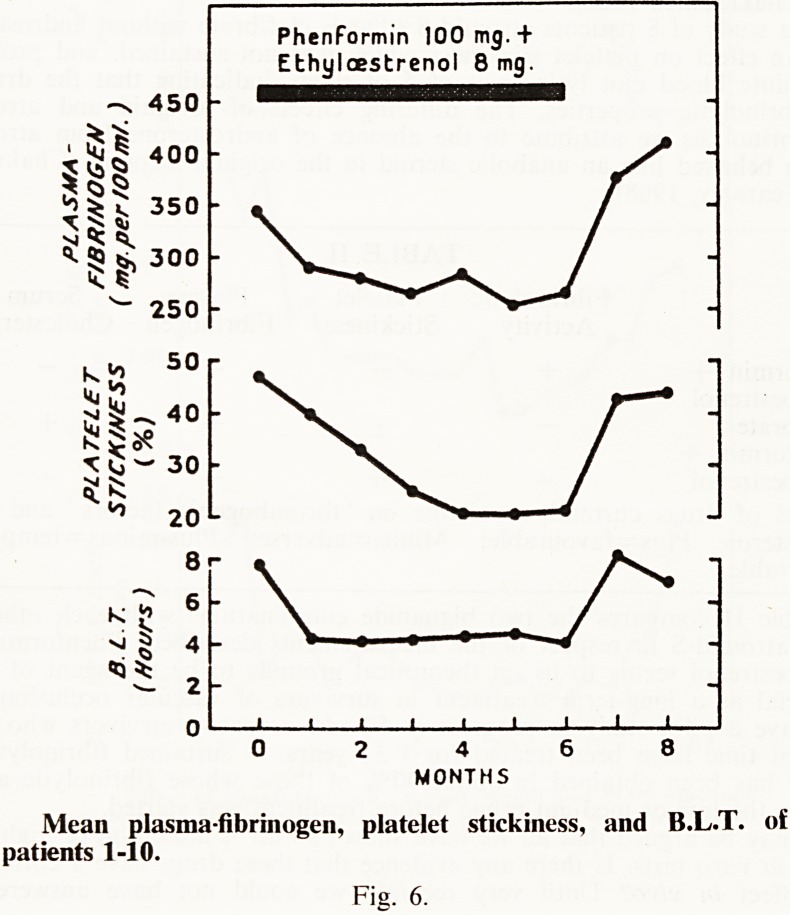


**Fig. 7. f7:**